# Preferred Panethnic Terms Among Latina/o and Hispanic Sexual and Gender Minority People

**DOI:** 10.1001/jamanetworkopen.2026.0060

**Published:** 2026-02-26

**Authors:** Alexis Ceja, Nguyen K. Tran, Juan M. Peña, David J. Kinitz, Devin Hursey, Ramon Ramirez, Lilia Cervantes, Micah E. Lubensky, Juno Obedin-Maliver, Annesa Flentje, Mitchell R. Lunn

**Affiliations:** 1 Department of Mathematics, California State University, Fullerton; 2 The PRIDE Study/PRIDEnet, Stanford University School of Medicine, Palo Alto, California; 3 Department of Obstetrics and Gynecology, Stanford University School of Medicine, Stanford, California; 4 Department of Psychology, College of Social Sciences, San José State University, San José, California; 5 College of Social Work, The Ohio State University, Columbus; 6 Division of Hospital Medicine and General Internal Medicine, Department of Medicine, University of Colorado School of Medicine, Aurora, Colorado; 7 Department of Epidemiology and Population Health, Stanford University School of Medicine, Stanford, California; 8 Stanford Prevention Research Center, Department of Medicine, Stanford University School of Medicine, Stanford, California; 9 Alliance Health Project, Department of Psychiatry and Behavioral Sciences, School of Medicine, University of California, San Francisco; 10 Division of Nephrology, Department of Medicine, Stanford University School of Medicine, Stanford, California

## Abstract

**Importance:**

Latino and Hispanic individuals in the US represent a diverse and growing population, including a significant number of sexual and gender minority (SGM) individuals whose identities challenge the use of a single panethnic (ie, ethnicity) label. Little is known about SGM individuals’ preferences for panethnic terms.

**Objective:**

To understand Latino and Hispanic SGM individuals’ preferences and rationales for panethnic terms.

**Design, Setting, and Participants:**

This cross-sectional study used survey data from SGM-identifying Latino and Hispanic adults in The PRIDE Study, an online, community-engaged cohort of SGM adults in the US completing 2021 or 2022 annual questionnaires, with qualitative analysis of an open-ended question asked to a subset about their preferred panethnic identification. Data analysis was conducted from June 2023 to November 2025.

**Main Outcomes and Measures:**

Survey responses for preferred panethnic terms and associated rationales.

**Results:**

Of 530 eligible participants, 517 reported their preferred panethnic term, and 376 provided their rationale. Among those who reported their panethnic term preferences, the median (IQR) age was 29.6 (24.2-40.5) years, among whom 253 (48.9%) identified as *Mexican* or *Mexican American*, 149 (28.8%) were gender diverse, 305 (59.0%) received a 4-year degree or higher, and 45 (8.7%) were born outside the US. *Latina* and *Latino* were the most preferred panethnic terms among 154 participants (29.8%), followed by *Hispanic* (126 participants [24.4%]), *Latinx* (86 participants [16.6%]), and *Latine* (47 participants [9.1%]) with others less commonly selected. A total of 59 (11.4%) selected another term or provided a write-in response for their panethnic identification. Six themes were identified in thematic analysis that reflected how preferred panethnic terms were shaped: (1) aligning with cultural norms, (2) influence of ethnoracial identity, (3) affirmation of gender and ethnicity, (4) linguistic and sociopolitical contexts, (5) delineating between Latin American and Spanish ancestry, and (6) cultural disconnection with origin group.

**Conclusions and Relevance:**

In this cross-sectional study with a qualitative analysis of an open-ended question, there was no clear consensus on preferred panethnic terms, and participants’ rationales indicated that personal, cultural, and linguistic factors related to identity and belonging influenced their preferences. Using panethnic terms in research, community engagement, and communication should be culturally relevant and context-specific.

## Key Points

**Question:**

What are Latino and Hispanic sexual and gender minority individuals’ preferences and rationales for using panethnic terms?

**Findings:**

In this cross-sectional study using survey data from 517 SGM participants, *Latina* or *Latino* were the most preferred panethnic terms (29.8%), followed by *Hispanic* (24.4%), *Latinx* (16.6%), and *Latine* (9.1%), with others less commonly selected. Among the 376 participants who provided a rationale for their preferences using write-in responses, preferences reflected complex personal, cultural, and linguistic considerations related to identity and belonging.

**Meaning:**

No clear consensus emerged regarding preferred panethnic terms, highlighting the need for a culturally informed, context-specific approach to using panethnic terms in research, community engagement, and communication.

## Teaser

This cross-sectional study evaluates Latina/o and Hispanic sexual and gender minority individuals’ preferences and rationale for panethnic terms.

## Introduction

Panethnic labels—terms used to group individuals from multiple ethnic, national, or cultural origins that may share certain sociocultural characteristics *(*eg, language, diet, religion, and geographic origin)^1,2^—are used in the US to classify populations. The US Census Bureau uses the panethnic label *Hispanic or Latino* to include individuals of Mexican, Puerto Rican, Cuban, Salvadoran, Dominican, Colombian, Guatemalan, and other Central or South American or other Spanish culture or origin.^3^ This panethnic identifier reflects institutional changes in federal data collection practices shaped by decades of community efforts to address undercounting of Latino and Hispanic populations and establish a more representative term for individuals of Latin American and Hispanic descent.^4,5,6,7^

Research on panethnic term preferences has largely focused on *Latino* or *Hispanic*. In national studies, *Hispanic* was often the most preferred term followed by *Latino*, though many reported having no preference between them.^8,9,10,11^ Multiple factors correlate with panethnic term preferences. In the 2013 National Survey for Latinos data, college graduates, those not of Mexican ethnicity, first- and second-generation immigrants, and respondents in the western US region were more likely to prefer *Latino* over *Hispanic*.^8^ This suggested that preferences may vary across Latino and Hispanic subgroups.

More recently, *Latinx* has emerged as an alternative. *Latinx* first appeared in the early 2000s within activist spaces to challenge the binary gendered grammatical system in Spanish and to be more inclusive of transgender and gender diverse (TGD) people (see eTable in Supplement 1 for definitions of key terms related to gender and ethnoracial identity).^12,13,14,15,16,17,18^ Between 2019 and 2023, awareness of *Latinx* increased by 24 percentage points among US Latino and Hispanic individuals, with 47% reporting having heard of *Latinx* in 2023.^11^ Its use in medical literature has also risen by 11 percentage points from 2016 to 2023.^19^ However, its adoption has been contentious. Critics of *Latinx* argue that it deviates from Spanish grammar and orthography and is an anglicized term that does not align with the community.^16,20^ Only 2% to 4% of Latino and Hispanic individuals prefer *Latinx*; however, preferences for *Latinx* were higher in certain subgroups, including those aged 18 to 29 years, college graduates, Democrats, Afro-Latinos, and individuals who identify as lesbian, gay, or bisexual.^10,11,21^ *Latine* has been proposed as a gender-inclusive alternative that is more aligned with Spanish grammar, though awareness is limited outside of Spanish-speaking TGD communities.^11,22^

The evolving landscape of panethnic terms reflects broader debates about identity, inclusion, and representation, particularly for sexual and gender minority (SGM) individuals (eg, lesbian, gay, bisexual, transgender, queer, intersex, and/or asexual) who have historically been excluded from research about their Latino/a and Hispanic community experiences. Studies that examine panethnic term preferences have rarely included SGM individuals or have not reported SGM-specific estimates. Consequently, little is known about how gender identity intersects with sociocultural experiences to shape panethnic term identification among SGM populations from Latin American and Hispanic backgrounds, which is important for inclusive research practices and public health communication strategies.^23,24^ Using survey data from a community-engaged study, we aimed to address this gap by identifying the preferred panethnic terms and underlying rationale among SGM individuals of Latin American and Hispanic descent.

## Methods

### Study Sample

Data for this cross-sectional study were from The PRIDE Study, an online, community-engaged, prospective cohort of SGM-identifying adults in the US and its territories who can participate in English.^25,26^ Details of The PRIDE Study^25^ and its national community engagement network, PRIDEnet,^26^ are described elsewhere. We pooled data from the lifetime and 2021 and 2022 annual health questionnaires to conduct a cross-sectional analysis of survey data with qualitative analysis of an open-ended question. We restricted the sample to a subset of participants who self-identified as *Hispanic*, *Latino*, or *Spanish* or provided write-in responses indicating this (eg, Brazilian). Participants provided electronic informed consent. Stanford University, University of California, San Francisco, and WIRB-Copernicus Group institutional review boards provided ethnics approval. The study followed the Strengthening the Reporting of Observational Studies in Epidemiology (STROBE) reporting guidelines.^27^

### Measures

#### Ethnoracial Identity

Participants self-selected 1 or more of the following identities: American Indian or Alaska Native; Asian; Black, African American, or African; Hispanic, Latino, or Spanish; Middle Eastern or North African; Native Hawaiian or other Pacific Islander; White; or none of these fully describe me (write-in option). Participants who selected Latino, Hispanic, or Spanish (hereafter, Latina/o and Hispanic) specified their ethnicity or ethnicities: Colombian, Cuban, Dominican, Ecuadorian, Honduran, Mexican or Mexican American, Puerto Rican, Salvadoran, Spanish, or none of these fully describe me (write-in option).

#### Panethnic Term Preference and Rationale

Latina/o and Hispanic participants were asked which single term “best describes [them] related to [their] Hispanic, Latino, or Spanish ethnicity.” Responses included: Chicana, Chicano, Hispanic, Hispano, Latina, Latine, Latino, Latinx, Spanish, or another term not listed (write-in option). Participants provided their reason(s) for their preferred term in response to an open-ended question: “You said [response] describes you best. If you wish, please tell us more about why you identify most with [response] and not the other terms listed.”

#### Sociodemographics

Sociodemographic information was obtained from the same annual questionnaire in which participants provided their preferred panethnic term. US Census region was determined by mapping self-reported zip code to the corresponding states. Nativity was assessed once in the lifetime questionnaire and categorized as US born or non–US born. For analysis by gender modality,^28^ we categorized participants into 3 groups: (1) cisgender participants, whose binary gender identity aligned with their assigned sex at birth; (2) transgender participants, whose binary gender identity did not align with their sex assigned at birth; and (3) gender diverse participants who endorsed a nonbinary identity (eg, agender, genderqueer, nonbinary, questioning, two-spirit, or a write-in response).

### Statistical Analysis

Descriptive statistics were used for the overall sample and by panethnic term preference. We calculated the percentage of self-reported preferred panethnic terms and categorized them into 7 groups (*Hispanic, Hispano, Latina/o, Latine, Latinx, Spanish*, and another term) to examine differences in sociodemographic characteristics across groups. Another term includes those who self-identified as *Chicana* or *Chicano* or provided a write-up response. Differences were assessed using χ^2^ or Fisher exact test for categorical data and Kruskal-Wallis test for continuous data. Statistical significance was determined with a 2-sided *P* < .05. All descriptive analyses were conducted in R version 4.3 (R Project for Statistical Computing).^29^ Data analysis was conducted from June 2023 to November 2025.

A thematic analysis was conducted on write-in responses to the open-ended question about preferred panethnic term to identify themes related to participants’ rationale for their preferred term.^30^ The coding team (A.C. and N.K.T.) inductively developed codes and used Dedoose version 9.0.17 (Sociocultural Research Consultants)^31^ to manage the data. The initial codebook was developed with a random 10% of the sample. The codebook was applied to the remaining responses with each coder independently coding half of the responses. Dependability and trustworthiness were supported by grounding coding in participants’ own words, writing analytic memos to reflect on conceptual issues, and meeting weekly to discuss coding inconsistencies and potential codebook modifications.^32^ Coding and analytic differences were resolved by reaching agreement among the coding team. Six themes were generated through this iterative process.

## Results

### Participants

Of 530 eligible participants, 517 reported their preferred panethnic term. The median (IQR) age was 29.6 (24.2-40.5) years (Table 1). About one-third (170 participants) exclusively self-identified as *Hispanic*, *Latino*, or *Spanish* as their ethnoracial identity. Of the 347 participants who did not exclusively self-identify as *Hispanic*, *Latino*, or *Spanish*, 13 (3.8%) identified as American Indian or Alaska Native; 6 (1.7%) identified as Asian; 14 (4.0%) identified as Black, African American, or African; 2 (0.6%) identified as Middle Eastern or North African; 227 (65.4%) identified as White, and 85 (24.5%) identified with more than 1 racial identity. The most frequently reported Latina/o and Hispanic ethnicity was Mexican or Mexican American (253 participants [48.9%]). Commonly endorsed gender identities included man (140 participants [27.1%]), cisgender woman (129 participants [25.0%]), and nonbinary (127 participants [24.6%]). A high percentage of participants reported having a 4-year degree or higher (305 participants [59.0%]), income below $50 000 (285 participants [55.1%]), being US-born (455 participants [88.0%]), and residence in the Western region of the US (202 participants [39.1%]).

**Table 1.** Participant Characteristics in the Overall Quantitative Sample and Qualitative Subset Used for Thematic Analysis

Characteristic	Participants, No. (%)	
	Overall sample (N = 517)	Qualitative subset (n = 376)
Age, median (IQR)	29.6 (24.2-40.5)	30.7 (24.9-42.8)
Ethnoracial identity		
American Indian or Alaska Native	13 (2.5)	12 (3.2)
Asian	6 (1.2)	4 (1.1)
Black, African American, or African	14 (2.7)	12 (3.2)
Hispanic, Latino, or Spanish only	170 (32.9)	117 (31.1)
Middle Eastern or North African	2 (0.4)	1 (0.3)
Native Hawaiian or other Pacific Islander	0	0
White	227 (43.9)	168 (44.7)
Selected more than 1 option^a^	85 (16.4)	62 (16.5)
Latina/o and Hispanic ethnicity		
Colombian	19 (3.7)	10 (2.7)
Cuban	38 (7.4)	27 (7.2)
Dominican	7 (1.4)	5 (1.3)
Ecuadorian	9 (1.7)	5 (1.3)
Honduran	6 (1.2)	6 (1.6)
Mexican or Mexican American	253 (48.9)	187 (49.7)
Puerto Rican	84 (16.2)	55 (14.6)
Salvadoran	12 (2.3)	10 (2.7)
Spanish	102 (19.7)	77 (20.5)
Another ethnicity	93 (18.0)	68 (18.1)
Gender identity^b^		
Agender	32 (6.2)	20 (5.3)
Cisgender man	118 (22.8)	96 (25.5)
Cisgender woman	129 (25.0)	96 (25.5)
Genderqueer	67 (13.0)	51 (13.6)
Man	140 (27.1)	103 (27.4)
Nonbinary	127 (24.6)	93 (24.7)
Questioning	32 (6.2)	27 (7.2)
Transgender man	75 (14.5)	46 (12.2)
Transgender woman	22 (4.3)	16 (4.3)
Two-spirit	15 (2.9)	11 (2.9)
Woman	113 (21.9)	80 (21.3)
Another gender identity	52 (10.1)	42 (11.2)
Gender modality		
Cisgender	268 (51.8)	198 (52.7)
Gender diverse	149 (28.8)	113 (30.1)
Transgender	100 (19.3)	65 (17.3)
Sexual orientation^b^		
Asexual	64 (12.4)	49 (13.0)
Bisexual	163 (31.5)	116 (30.9)
Gay	195 (37.7)	147 (39.1)
Lesbian	94 (18.2)	69 (18.4)
Pansexual	99 (19.1)	74 (19.7)
Queer	204 (39.5)	147 (39.1)
Questioning	20 (3.9)	15 (4.0)
Same-gender loving	39 (7.5)	33 (8.8)
Straight or heterosexual	18 (3.5)	12 (3.2)
Two-spirit	9 (1.7)	7 (1.9)
Another sexual orientation	22 (4.3)	16 (4.3)
Education level		
High school or GED graduate or less	27 (5.2)	18 (4.8)
Trade school, some college, or 2-y degree	112 (21.7)	83 (22.1)
4-y degree	152 (29.4)	121 (32.2)
Graduate degree	153 (29.6)	118 (31.4)
Missing	73 (14.1)	36 (9.6)
Annual individual income		
$0-20 000	148 (28.6)	107 (28.5)
$20 001-50 000	137 (26.5)	107 (28.5)
$50 001-100 000	100 (19.3)	76 (20.2)
≥$100 001	56 (10.8)	48 (12.8)
Missing	76 (14.7)	38 (10.1)
Nativity		
US born	455 (88.0)	334 (88.8)
Non–US born	45 (8.7)	30 (8.0)
Missing	17 (3.3)	12 (3.2)
US census region		
Northeast	94 (18.2)	73 (19.4)
Midwest	65 (12.6)	42 (11.2)
South	142 (27.5)	106 (28.2)
West	202 (39.1)	151 (40.2)
Missing	14 (2.7)	4 (1.1)

Abbreviation: GED, General Educational Development.

^a^ Inclusive of participants who identified as Hispanic, Latino, or Spanish in addition to 1 or more ethnicities or races.

^b^ Participants were able to select multiple options, thus, the sum of percentages may be greater than 100%.

*Latina* or *Latino* (154 participants [29.8%]) were the most frequently preferred panethnic terms (56 *Latina* and 98 *Latino*), followed by *Hispanic* (126 participants [24.4%]), *Latinx* (86 participants [16.6%]), another term (59 participants [11.4%]), *Latine* (47 participants [9.1%]), *Spanish* (36 participants [7.0%]), and *Hispano* (9 participants [1.7%]) (Table 1; eFigure in Supplement 1). Among those who identified with another term, the most common responses were *Chicano* (18 participants [30.5%]), *Chicana* (13 participants [22.0%]), and *Mexican* or *Mexican American* (7 participants [11.9%]) (eFigure in Supplement 1).

Term preference varied by age, gender identity, gender modality, and sexual orientation (Table 2). Participants who preferred *Latine* or *Latinx* were more likely to be younger, self-identify as gender diverse (including agender, genderqueer, and nonbinary), and self-identify as queer compared with those who preferred a different term. There were nonsignificant differences by ethnoracial groups, education level, income, nativity, and region.

**Table 2.** Characteristics of the Overall Sample Stratified by Preferred Panethnic Term (N = 517)

Characteristic	Participants, No. (%)							*P* value
	Hispanic (n = 126)	Hispano (n = 9)	Latina/o (n = 154)	Latine (n = 47)	Latinx (n = 86)	Spanish (n = 36)	Another term (n = 59)^a^	
Age, median (IQR)	29.7 (24.3-40.8)	34.6 (26.7-40.9)	30.3 (25.7-40.5)	25.4 (21.8-29.8)	28.4 (23.1-35.6)	38.0 (27.2-47.1)	34.1 (23.7-45.9)	<.001
Ethnoracial identity								
American Indian or Alaska Native	2 (1.6)	0	3 (1.9)	1 (2.1)	2 (2.3)	0	5 (8.5)	.23
Asian	2 (1.6)	0	2 (1.3)	0	1 (1.2)	0	1 (1.7)	
Black, African American or African	2 (1.6)	0	7 (4.5)	2 (4.3)	3 (3.5)	0	0	
Hispanic, Latino, or Spanish only	39 (31.0)	6 (66.7)	59 (38.3)	20 (42.6)	27 (31.4)	4 (11.1)	15 (25.4)	
Middle Eastern or North African	1 (0.8)	0	1 (0.6)	0	0	0	0	
Native Hawaiian or other Pacific Islander	0	0	0	0	0	0	0	
White	62 (49.2)	2 (22.2)	58 (37.7)	17 (36.2)	39 (45.3)	24 (66.7)	25 (42.4)	
Selected more than 1 option^a^	18 (14.3)	1 (11.1)	24 (15.6)	7 (14.9)	14 (16.3)	8 (22.2)	13 (22.0)	
Latina/o and Hispanic ethnicity								
Colombian	3 (2.4)	1 (11.1)	11 (7.1)	2 (4.3)	2 (2.3)	0	0	.08
Cuban	8 (6.3)	0	14 (9.1)	4 (8.5)	9 (10.5)	0	3 (5.1)	.40
Dominican	0	0	2 (1.3)	2 (4.3)	3 (3.5)	0	0	.17
Ecuadorian	5 (4.0)	0	2 (1.3)	1 (2.1)	0	1 (2.8)	0	.34
Honduran	1 (0.8)	0	2 (1.3)	0	3 (3.5)	0	0	.42
Mexican or Mexican American	68 (54.0)	4 (44.4)	67 (43.5)	23 (48.9)	42 (48.8)	6 (16.7)	43 (72.9)	<.001
Puerto Rican	22 (17.5)	2 (22.2)	29 (18.8)	12 (25.5)	15 (17.4)	0	4 (6.8)	.02
Salvadoran	4 (3.2)	0	6 (3.9)	1 (2.1)	0	1 (2.8)	0	.45
Spanish	27 (21.4)	2 (22.2)	16 (10.4)	3 (6.4)	17 (19.8)	32 (88.9)	5 (8.5)	<.001
Another ethnicity	16 (12.7)	3 (33.3)	33 (21.4)	8 (17.0)	19 (22.1)	0	14 (23.7)	.02
Gender identity^b^								
Agender	6 (4.8)	1 (11.1)	4 (2.6)	9 (19.1)	6 (7.0)	3 (8.3)	3 (5.1)	.01
Cisgender man	33 (26.2)	3 (33.3)	55 (35.7)	2 (4.3)	8 (9.3)	6 (16.7)	11 (18.6)	<.001
Cisgender woman	33 (26.2)	2 (22.2)	38 (24.7)	6 (12.8)	23 (26.7)	7 (19.4)	20 (33.9)	.31
Genderqueer	13 (10.3)	2 (22.2)	7 (4.5)	16 (34.0)	18 (20.9)	2 (5.6)	9 (15.3)	<.001
Man	44 (34.9)	5 (55.6)	48 (31.2)	5 (10.6)	14 (16.3)	11 (30.6)	13 (22.0)	.002
Nonbinary	20 (15.9)	2 (22.2)	10 (6.5)	34 (72.3)	39 (45.3)	6 (16.7)	16 (27.1)	<.001
Questioning	8 (6.3)	1 (11.1)	6 (3.9)	8 (17.0)	5 (5.8)	0	4 (6.8)	.03
Transgender man	16 (12.7)	1 (11.1)	19 (12.3)	11 (23.4)	16 (18.6)	6 (16.7)	6 (10.2)	.40
Transgender woman	3 (2.4)	0	9 (5.8)	0	5 (5.8)	1 (2.8)	4 (6.8)	.41
Two-spirit	3 (2.4)	0	3 (1.9)	1 (2.1)	4 (4.7)	3 (8.3)	1 (1.7)	.42
Woman	28 (22.2)	2 (22.2)	29 (18.8)	5 (10.6)	17 (19.8)	14 (38.9)	18 (30.5)	.04
Another gender identity	10 (7.9)	2 (22.2)	8 (5.2)	8 (17.0)	12 (14.0)	2 (5.6)	10 (16.9)	.03
Gender modality								
Cisgender	76 (60.3)	6 (66.7)	102 (66.2)	5 (10.6)	26 (30.2)	21 (58.3)	32 (54.2)	<.001
Gender diverse	31 (24.6)	2 (22.2)	22 (14.3)	31 (66.0)	39 (45.3)	7 (19.4)	17 (28.8)	
Transgender	19 (15.1)	1 (11.1)	30 (19.5)	11 (23.4)	21 (24.4)	8 (22.2)	10 (16.9)	
Sexual orientation^b^								
Asexual	19 (15.1)	1 (11.1)	13 (8.4)	5 (10.6)	14 (16.3)	7 (19.4)	5 (8.5)	.33
Bisexual	38 (30.2)	3 (33.3)	38 (24.7)	20 (42.6)	29 (33.7)	15 (41.7)	20 (33.9)	.23
Gay	56 (44.4)	4 (44.4)	75 (48.7)	9 (19.1)	21 (24.4)	14 (38.9)	16 (27.1)	<.001
Lesbian	21 (16.7)	0	26 (16.9)	7 (14.9)	14 (16.3)	9 (25.0)	17 (28.8)	.20
Pansexual	26 (20.6)	2 (22.2)	21 (13.6)	12 (25.5)	21 (24.4)	7 (19.4)	10 (16.9)	.40
Queer	40 (31.7)	3 (33.3)	46 (29.9)	27 (57.4)	47 (54.7)	13 (36.1)	28 (47.5)	<.001
Questioning	5 (4.0)	0	2 (1.3)	1 (2.1)	5 (5.8)	4 (11.1)	3 (5.1)	.14
Same-gender loving	10 (7.9)	0	7 (4.5)	4 (8.5)	11 (12.8)	3 (8.3)	4 (6.8)	.39
Straight or heterosexual	3 (2.4)	1 (11.1)	5 (3.2)	0	2 (2.3)	4 (11.1)	3 (5.1)	.10
Two-spirit	1 (0.8)	0	2 (1.3)	0	3 (3.5)	3 (8.3)	0	.04
Another sexual orientation	2 (1.6)	1 (11.1)	7 (4.5)	1 (2.1)	4 (4.7)	3 (8.3)	4 (6.8)	.40
Education level								
High school or GED graduate or less	8 (6.3)	1 (11.1)	11 (7.1)	3 (6.4)	2 (2.3)	2 (5.6)	0	.49
Trade school, some college, or 2-y degree	30 (23.8)	4 (44.4)	27 (17.5)	9 (19.1)	17 (19.8)	11 (30.6)	14 (23.7)	
4-y degree	38 (30.2)	1 (11.1)	38 (24.7)	17 (36.2)	28 (32.6)	11 (30.6)	19 (32.2)	
Graduate degree	28 (22.2)	2 (22.2)	54 (35.1)	13 (27.7)	27 (31.4)	10 (27.8)	19 (32.2)	
Missing	22 (17.5)	1 (11.1)	24 (15.6)	5 (10.6)	12 (14.0)	2 (5.6)	7 (11.9)	
Annual individual income								
$0-20 000	36 (28.6)	3 (33.3)	45 (29.2)	18 (38.3)	20 (23.3)	13 (36.1)	13 (22.0)	.40
$20 001-50 000	30 (23.8)	3 (33.3)	32 (20.8)	15 (31.9)	32 (37.2)	7 (19.4)	18 (30.5)	
$50 001-100 000	26 (20.6)	1 (11.1)	30 (19.5)	8 (17.0)	14 (16.3)	6 (16.7)	15 (25.4)	
≥$100 001	12 (9.5)	1 (11.1)	22 (14.3)	1 (2.1)	7 (8.1)	7 (19.4)	6 (10.2)	
Missing	22 (17.5)	1 (11.1)	25 (16.2)	5 (10.6)	13 (15.1)	3 (8.3)	7 (11.9)	
Nativity								
US born	114 (90.5)	7 (77.8)	128 (83.1)	44 (93.6)	75 (87.2)	35 (97.2)	52 (88.1)	.12
Non–US born	7 (5.6)	2 (22.2)	22 (14.3)	3 (6.4)	6 (7.0)	1 (2.8)	4 (6.8)	
Missing	5 (4.0)	0	4 (2.6)	0	5 (5.8)	0	3 (5.1)	
US census region								
Northeast	25 (19.8)	4 (44.4)	16 (10.4)	11 (23.4)	22 (25.6)	5 (13.9)	11 (18.6)	.14
Midwest	13 (10.3)	2 (22.2)	18 (11.7)	10 (21.3)	12 (14.0)	4 (11.1)	6 (10.2)	
South	38 (30.2)	2 (22.2)	50 (32.5)	12 (25.5)	20 (23.3)	9 (25.0)	11 (18.6)	
West	47 (37.3)	1 (11.1)	64 (41.6)	13 (27.7)	31 (36.0)	18 (50.0)	28 (47.5)	
Missing	3 (2.4)	0	6 (3.9)	1 (2.1)	1 (1.2)	0	3 (5.1)	

Abbreviation: GED, General Educational Development.

^a^ Inclusive of participants who identified as Hispanic, Latino, or Spanish in addition to 1 or more ethnicities or races.

^b^ Participants were able to select multiple options, thus, the sum of percentages may be greater than 100%.

### Themes

Write-in responses were provided by 376 participants. Participants identified the complexity of Latina/o and Hispanic panethnic identity in the US with responses such as “it’s complicated” or “I am not sure.” We analyzed this complexity through the themes presented in the following sections with additional quotes and participant characteristics shown in Table 3.

**Table 3.** Themes and Example Quotes of Participants’ Rationale for Their Preferred Panethnic Term (N = 376)

Themes	Example quotes (ethnicity or ethnicities), age decade, gender identity or identities, and preferred panethnic term)
1. Aligning with cultural norms	“It’s a title that describes my comfort zone in culture and heritage.” (Mexican or Mexican American, 60s, transgender woman, Latina) “It’s what my family used growing up.” (Mexican or Mexican American, 20s, genderqueer, nonbinary, Hispanic) “Coming from south Texas with a direct connection to Mejico, activists from this area use Chicana.” (Mexican or Mexican American, 60s, cisgender woman, Chicana) “I’m comfortable with the term, being the one I’ve used the longest. But I’m also not opposed to the other terms.” (Mexican or Mexican American, 30s, cisgender man, Latino)
2. Influence of ethnoracial identity	“I have Spanish ancestors, but not South or Central American ancestors…I feel it would be disrespectful to both the struggles and culture of Latin American people to call myself by any of the other terms.” (Spanish, 20s, transgender woman, woman, Spanish) “Latin heritage is important to me, and Hispanic is not.” (Mexican or Mexican American, 40s, cisgender man, Latino) “My family has been in Texas since before it was a part of the United States and was [its] own republic. We are ethnically Mexican, but none of my family has ever immigrated here.” (Mexican or Mexican American, 20s, agender, Chicano) “It’s what I’ve always felt fits best because I am part of two Hispanic cultures, Puerto Rican through my dad and Spanish through my mom.” (Puerto Rican, Spanish, 30s, nonbinary, another gender, Hispanic) “It describes a larger cultural identity based on this continent. It also has come to embrace various Indigenous identities (unlike Hispanic, that usually is seen as relating to Spanish/European culture and ancestry).” (Spanish, another ethnicity, 50s, cisgender man, Latino) “As a Black Latina, my experience with Latinidad differs significantly from White or Brown Latinas, because I am Black before I am Latina both in the eyes of myself and my family and in the eyes of people who would (or would not) categorize me as Latina.” (Another ethnicity [Panamanian], 20s, cisgender woman, woman, another term [Afrolatina])
3. Affirmation of gender and ethnicity	“I identify as male, so using the male-gendered version of Latino/Latina/Latine works best for me…” (Puerto Rican, 20s, man, transgender man, Latino) “As I identify as a cisgendered woman, Latina encompasses that as well as being born in the US with heritage from Spanish-speaking countries, including Spain.” (Salvadoran, Spanish, another ethnicity, 30s, cisgender woman, Latina) “I am half Mexican but am also nonbinary; Latine best describes where I fit within the diaspora.” (Mexican, 30s, agender, genderqueer, nonbinary, Latine)
4. Linguistic and Socio-political contexts	“I understand Hispanic to be ‘of Spanish-speaking origin’, and I do speak Spanish.” (Puerto Rican, 20s, cisgender woman, woman, Hispanic) “I was born and raised in Colombia and Spanish is my first language, therefore I identify more as Hispano.” (Colombian, 30s, cisgender man, Hispano) “I find the term ‘Latinx’ extremely insulting and disrespectful to the Spanish language and the cultures and people that speak it, as it adds a nonnative construction to a Spanish word...” (Cuban, 20s, agender, cisgender woman, nonbinary, another gender, Latina) “I liked Latinx but I prefer Latine because it accomplishes the intentional neutrality that Latinx does but without the very white influence of the x.” (Cuban, 20s, man, transgender man, Latine) “I feel good with Latino and Latina and use those around non-LGBT Latinos. Privately, or around trans/ally folks, I prefer Latinx to reflect my agender experiences...” (Another ethnicity, 20s, agender, genderqueer, nonbinary, questioning, another gender, Latinx) “… Latinx centers the mestizaje that created Latin America as we know it, whereas Hispanic centers colonial roots…” (Cuban, 20s, genderqueer, nonbinary, another gender, Latinx) “Latinx/Mexican is more what I would describe only because Hispanic is a derogatory word used to describe people of my background, given to us by colonizers.” (Mexican or Mexican American, 20s, man, transgender man, Latino)
5. Delineating between Latin American and Spanish ancestry	“Though the term ‘Hispanic’ would also describe me, I like Latino or Mexican because it distances the Spanish colonizers from me and my families’ identity.” (Mexican or Mexican American, 20s, transgender man, Latino) “People treat ‘Hispanic’ as South American but exclude Brazil.” (Another ethnicity [Brazilian], 20s, cisgender woman, Latina)
6. Cultural disconnection with origin group	“I’m 1st generation American; my parents were both Cuban and arrived in the US as older teens/young adults. I’m neither viewed as American in the US, not Cuban in Cuba (never visited).” (Cuban, another ethnicity, 40s, cisgender woman, woman, another term [Cuban]) “I grew up whitewashed because my family didn’t want us to have the same struggles, so I don’t have a strong Hispanic identity.” (Puerto Rican, 30s, transgender woman, Hispanic) “I am half Puerto Rican but don’t speak Spanish so it’s really more of an ethnic background than an identity for me.” (Puerto Rican, 50s, woman, Hispanic)

Abbreviation: LGBT, lesbian, gay, bisexual, or transgender.

#### Aligning With Cultural Norms

Participants described the selection of panethnic identity labels based on long-standing usage, familial influence, geographical context, and activist traditions within specific communities, which resulted in participants’ personal comfort and acceptance by their community. Participants gravitated toward terms that resonated with their lived experiences and cultural environments, even as they remained open to other panethnic designations. For instance, participants who preferred *Latina/o* selected terms that aligned with their personal sense of cultural identity. One participant described their chosen term as “a title that describes my comfort zone in culture and heritage.” Family norms, local customs, and regional influences also played an important role in shaping preferences. One participant stated that they chose *Hispanic* because “It’s what my family used growing up,” while another participant explained their preference for *Chicana* as it relates to the activist tradition of their region: “Coming from south Texas with a direct connection to Mejico, activists from this area use Chicana.” Although participants expressed strong attachments to their preferred labels, they conveyed openness to alternatives, suggesting that preferences could evolve.

#### Influence of Ethnoracial Identity

Participants described navigating the intersections of their racial identity, Indigenous heritage, mixed ancestry, and generational ties to specific geographies. This illustrated how participants reconcile or differentiate between cultural influences and position themselves within broader ethnoracial categories in relation to their Latina/o and Hispanic identity. One Spanish participant stated, “I have Spanish ancestors, but not South or Central American ancestors...I feel it would be disrespectful to both the struggles and culture of Latin American people to call myself by any of the other terms.” In contrast, participants who preferred *Latina/o* valued the emphasis on their Latin American heritage. Those who preferred *Chicana* or *Chicano* highlighted their Mexican heritage and its connection to regions such as Texas before US colonization: “My family has been in Texas since before it was a part of the United States and was [its] own republic. We are ethnically Mexican, but none of my family has ever immigrated here.” Multicultural identities also shaped preferences with some participants favoring *Hispanic* because it encompassed their multiple ethnicities and cultures: “It’s what I’ve always felt fits best because I am part of two Hispanic cultures, Puerto Rican through my dad and Spanish through my mom.” Others favored *Latina/o* for its recognition of Indigenous ancestry and its broader association with the Americas, which they felt were missing from the term *Hispanic*. The intersection of race and ethnicity was particularly salient for Black participants. One participant explained her preference for “*Afro-Latina*” (provided as a write-in response), “As a Black Latina, my experience with Latinidad differs significantly from White or Brown Latinas, because I am Black before I am Latina both in the eyes of myself and my family and in the eyes of people who would (or would not) categorize me as Latina.” This highlights how racial dynamics shaped feelings of inclusion in Latinidad and how panethnic terms may inadequately represent the experiences of Black individuals.

#### Affirmation of Gender and Ethnicity

Participants described how chosen terms aligned with their gender expression while also acknowledging their cultural heritage. For some, using a gendered term that aligned with their gender identity, such as *Latina/o*, felt most appropriate. This was true for transgender and cisgender individuals alike who found that gendered terms reflected their lived experiences. One Puerto Rican transgender man explained, “I identify as male, so using the male-gendered version of Latino/Latina/Latine works best for me,” highlighting how the masculine form affirms his gender identity. Gender diverse participants frequently preferred *Latine*, which they felt better captured their position within the broader Latin American diaspora.

#### Linguistic and Sociopolitical Contexts

Participants discussed how their panethnic identification was influenced by language proficiency, linguistic traditions, and sociopolitical environments that contribute to perceptions of SGM acceptance. Individuals who identified as bilingual in their responses felt a strong connection to their heritage through language, preferring panethnic terms such as *Hispanic* or *Hispano*. This theme captured debates around language evolution and authenticity. Some expressed frustrations with *Latinx* because of its anglicized influence, with one participant describing it as “extremely insulting and disrespectful to the Spanish language.” Others, however, embraced newer terms like *Latine* for their perceived neutrality without “White influence.”

These linguistic preferences often intersected with sociopolitical considerations. Participants framed the use of *Latinx* as a form of political resistance against colonial labels and an affirmation of their cultural roots. One participant explained, “Latinx centers the mestizaje that created Latin America as we know it, whereas Hispanic centers colonial roots.” This sentiment was echoed by those who preferred *Latina/o* and expressed strong opposition to the term *Hispanic*, describing it as “derogatory” and viewing it as imposed by colonizers. In addition, participants reflected how their social environment influenced their use of panethnic terms. One agender participant described how they modify their terminology based on their audience: “I feel good with Latino and Latina and use those around non-LGBT Latinos. Privately, or around trans/ally folks, I prefer Latinx to reflect my agender experiences.”

#### Delineating Between Latin American and Spanish Ancestry

Participants emphasized the nuanced distinctions between their Latin American heritage and Spanish colonial history and the complexities of defining Latin American identity for those who were not colonized by Spain. These participants often included those who preferred *Latina/o* or a specific ethnicity. One Mexican American participant stated, “I like Latino or Mexican because it distances the Spanish colonizers from me and my families’ identity.” A Brazilian participant noted, “People treat ‘Hispanic’ as South American but exclude Brazil,” pointing out the limitations of certain terms in representing the full diversity of Latin American experiences. Others with Spanish ancestry also felt it was inappropriate to use labels associated with Latin American experiences out of respect for those distinct histories and cultures.

#### Cultural Disconnection With Origin Group

Participants who were born in the US described feelings of detachment and distance from their cultural heritage and how it shaped their panethnic identification, often grappling with belonging. For these participants, feelings of disconnection stemmed from assimilation pressures and limited exposure to their cultural heritage. One participant described that while they chose *Hispanic*, they did not strongly connect with their Hispanic identity since they grew up “whitewashed.” This disconnection led to shifts in self-perception and greater uncertainty about one’s cultural identity. Participants noted feeling caught between 2 cultures—neither fully accepted in their country of origin nor in the US. One participant explained, “I’m 1st generation American; my parents were both Cuban and arrived in the US as older teens/young adults. I’m neither viewed as American in the US, not Cuban in Cuba (never visited).”

## Discussion

This study examined panethnic term preferences and underlying rationale among Latina/o and Hispanic SGM participants. Although *Latina/o* emerged as the most frequently endorsed term (28.9%), followed by *Hispanic*, there was no clear consensus. This is contrary to prior work suggesting that *Hispanic* is the preferred panethnic term to describe individuals of Latin American or Hispanic origin.^10,11^ About 10% of participants did not identify with any of the prespecified panethnic terms. Instead, they provided terms that were specific to their heritage (eg, *Mexican*, *Cuban*, and *Chicano*), consistent with other survey data.^33^ Several themes influenced participants’ preferences for panethnic terms, reflecting the interplay of cultural norms, ethnoracial identity, and language in affirming their gender and ethnic identities. Given the current national discourse regarding panethnic terminology among Latino/a and Hispanic communities,^10,11^ these findings provide timely guidance and highlight the need for a nuanced, context-sensitive approach to using panethnic terms in research, community engagement, and health communication among Latino/a and Hispanic SGM individuals and the broader Latino/a and Hispanic community.

Building on prior research,^8,10,11,21^ our findings highlight the heterogeneity of panethnic term preferences among Latina/o and Hispanic SGM individuals. The preference for *Latinx* and *Latine* among younger, queer, and gender diverse participants aligns with previous research.^11,21^ This may reflect changing attitudes toward gender inclusivity and a growing awareness of the limitations of binary gendered language. However, we found that 32% of participants who preferred *Latinx* were cisgender, and 27% of those who preferred *Hispanic* were gender diverse. This challenges assumptions of *Latinx* and *Hispanic* as panethnic identifiers and suggests that *Latinx* may not be primarily preferred by gender diverse individuals, and the use of *Hispanic* may not be seen as exclusionary by all gender diverse people.

The qualitative findings contextualize the quantitative results by highlighting how lived experiences shaped panethnic term preferences. Among transgender participants, similarities in term preferences may reflect perceptions that *Latina/o*, *Latinx*, or *Latine* can be affirming of both their gender identity and ethnicity. Gender diverse participants preferred *Latinx* because of its deliberate queering (eTable in Supplement 1 for definition) of binary gendered language. However, use of panethnic term changed depending on the audience and/or context: participants selected *Latinx* in English-speaking or more SGM-supportive environments while choosing other terms for easier communication and/or safety in less-affirming spaces. Despite concerns about linguistic accessibility, many valued *Latinx* for centering political resistance and gender diverse representation.^17,18,20^ Additionally, views on the origins of panethnic terms varied; some saw terms like *Hispanic* as colonial impositions, while others found legitimacy in their Spanish linguistic roots. This tension has been previously discussed,^2,8^ and our results underscore the complexity of panethnic identity formation and the contested meanings ascribed to language within Latina/o and Hispanic SGM communities.

Using panethnic terms may not be appropriate or effective in all contexts. Many participants preferred their specific ethnicity over a panethnic label, as the latter may overlook the distinct experiences of Latina/o and Hispanic SGM individuals, particularly among Black and Indigenous communities. This emphasizes the importance of intersectionality in health equity research focusing on Latina/o and Hispanic ethnoracial identities^34,35^ and the need to use multidimensional measures of ethnoracial identity and/or allowing individuals to self-define their identity through qualitative responses.^36^ Defaulting to panethnic terms in research risks masking important cultural, socioeconomic, and health differences, especially for individuals whose identities do not align with such terms.^6,15,23,37^ Researchers working with SGM communities should consider collecting and reporting data on both panethnic and specific ethnic identities (eg, Puerto Rican, Cuban, and Salvadoran) to accurately represent the community.^14,15,37^ Community-engaged partnerships can inform how best to describe Latina/o and Hispanic SGM populations, taking into account the research goals, data collection context, and the preferences of those being represented.

Our results have important implications for health communication and addressing health inequities among Latina/o and Hispanic SGM populations, particularly for populations with intersectional identities. In health care settings, using preferred identity labels may foster trust, improve patient-clinician communication, and enhance culturally competent care.^38^ Healthcare practitioners should receive training in inclusive practices, such as collecting comprehensive data, developing personalized care plans that acknowledge the individuals’ intersectional experiences, and ensuring inclusive communication strategies, especially using preferred labels. These efforts will help create a more inclusive health care environment, reducing health care avoidance and improving retention.

### Limitations

This study has limitations. Participants were primarily US-born who understood English, were well-educated, and self-identified as *Latino*, *Hispanic*, or *Spanish* in the questionnaire. Nearly half of participants were of Mexican descent and more than one-third resided in the Western states. Thus, our results may not generalize to all Latino/a and Hispanic SGM communities. The PRIDE Study did not capture primary spoken language aside from self-report in write-in responses, thus we could not explore differences in preferred panethnic terms and rationale by primary language. Specific generational contexts at the time of data collection may have influenced term preference^39^; however, we lack quantitative data to show how these factors influenced participants’ self-identification. Finally, write-in responses may lack the depth that interviews or focus groups can offer. Future research should consider in-depth interviews over multiple time points to examine how acceptance of specific panethnic terms across intersecting identities (eg, gender, age, nativity, and region) evolve over time.

## Conclusions

In this cross-sectional study with qualitative analyses of an open-ended question, we found that panethnic labels play an important role in fostering collective visibility and political solidarity, but they risk obscuring the diverse experiences within Latina/o and Hispanic SGM communities.^2^ Continued community-engaged research is needed to refine terminology and improve data collection practices that are inclusive and reflective of diverse lived experiences for SGM people. Such efforts are essential for advancing equity in research, policy, and public discourse.

## Notes

Supplement 1. eTable. Definition of Key Terms eFigure. Response Frequency of (a) Self-Identified Panethnic Term Among the Overall Quantitative Sexual and Gender Minority Sample (n=517) and (b) of Self-Reported or Write-in Responses Among Those Identified With “Another Term” (n=59) eReferences.

Supplement 2. Data Sharing Statement
