# Supplementary material for: Preferred Panethnic Terms Among Latina/o and Hispanic Sexual and Gender Minority People
Source: JAMA Netw Open. 2026 Feb 26;9(2):e260060. doi: 10.1001/jamanetworkopen.2026.0060 (PMC12947012; doi:10.1001/jamanetworkopen.2026.0060)
Supplement: Supplement 1. — eTable. Definition of Key Terms eFigure. Response Frequency of (a) Self-Identified Panethnic Term Among the Overall Quantitative Sexual and Gender Minority Sample (n=517) and (b) of Self-Reported or Write-in Responses Among Those Identified With “Another Term” (n=59) eReferences. [file jamanetwopen-e260060-s001.pdf]

Supplemental Online Content

Ceja A, Tran NK, Peña JM, et al. Preferred Panethnic Terms Among Latina/o and Hispanic Sexual and Gender Minority People. *JAMA Netw Open*. 2026;9(2):e260060. doi:10.1001/jamanetworkopen.2026.0060

eTable. Definition of Key Terms

eFigure. Response Frequency of (a) Self-Identified Panethnic Term Among the Overall Quantitative Sexual and Gender Minority Sample (n=517) and (b) of Self-Reported or Write-in Responses Among Those Identified With “Another Term” (n=59)

eReferences

This supplemental material has been provided by the authors to give readers additional information about their work.

© 2026 Ceja A et al. *JAMA Network Open*.

eTable. Definition of key terms.

Term	Definition
Chicana/o	A term primarily used in the Southwestern United States to describe individuals of Mexican American descent. It originated from the Chicano Power movement and represents a distinct cultural and political identity.[1]
Gender identity	Personally held sense of one's gender as man/boy, woman/girl, another cultural gender, nonbinary, etc.[2]
Gender diverse	An umbrella term designed to be inclusive of a wide range of gender identities including those that transcend the spectrum of woman and man.[3]
Ethnoracial	Term relating to the social constructs and race and ethnicity that was designed to encompass diverse groups of people based on phenotype such as Latino.[4]
Queering	Method designed to challenge the heteronormativity of categories of binary identity categories in film, literature, and other forms of media[5]
Sex assigned at birth	Categories recorded on initial birth certificate; generally based on genital phenotype.[2]

© 2026 Ceja A et al. *JAMA Network Open*.

eFigure. Response frequency of (a) self-identified pan-ethnic term among the overall quantitative sexual and gender minority sample (n=517) and (b) of self-reported or write-in responses among those who identified with "another term" (n=59).

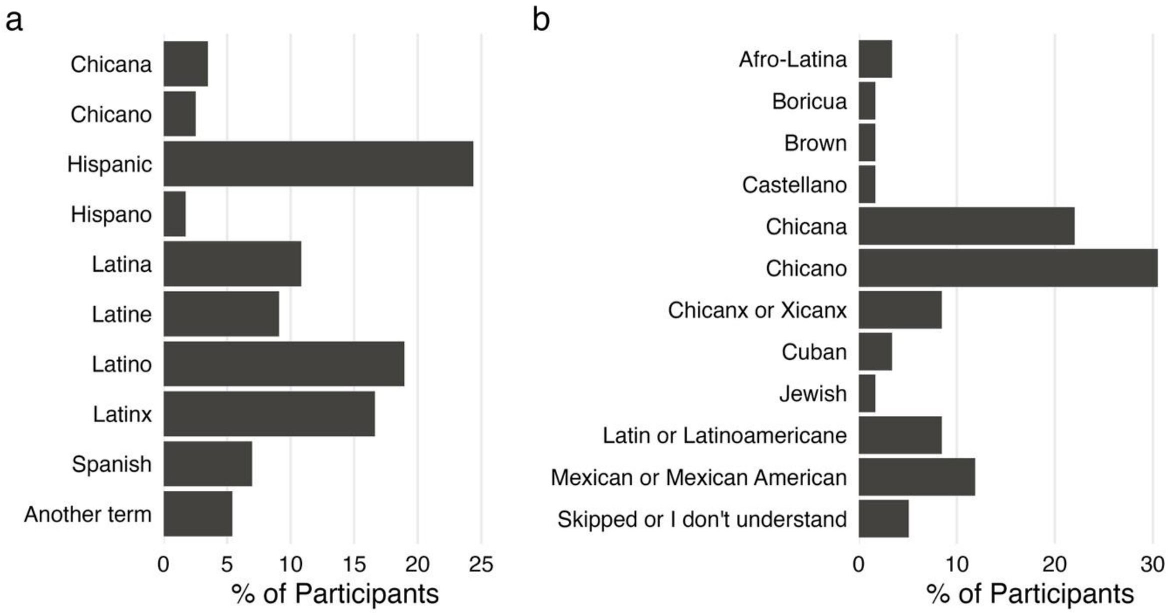

© 2026 Ceja A et al. *JAMA Network Open*.

eReferences

1. Contreras, Sheila Marie. 9. Chicana, Chicano, Chican@, Chicanx. In: *Keywords for Latina/o Studies*. New York University Press; 2017:32-35. doi:10.18574/nyu/9781479892532.003.0013

2. Bauer GR. Sex and Gender Multidimensionality in Epidemiologic Research. *Am J Epidemiol*. 2023;192(1):122-132. doi:10.1093/aje/kwac173

3. What is Gender Diversity? A Gender Agenda. Accessed November 26, 2025. https://genderrights.org.au/information-hub/what-is-gender-diversity/

4. Jiménez TR, Fields CD, Schachter A. How Ethnoraciality Matters: Looking inside Ethnoracial "Groups." *Social Currents*. 2015;2(2):107-115. doi:10.1177/2329496515579765

5. Cohen CJ. Punks, Bulldaggers, and Welfare Queens: The Radical Potential of Queer Politics? *GLQ: A Journal of Lesbian and Gay Studies*. 1997;3(4):437-465. doi:10.1215/10642684-3-4-437

© 2026 Ceja A et al. *JAMA Network Open*.
