# Supplementary material for: Preferred Panethnic Terms Among Latina/o and Hispanic Sexual and Gender Minority People
Source: JAMA Netw Open. 2026 Feb 26;9(2):e260060. doi: 10.1001/jamanetworkopen.2026.0060 (PMC12947012; doi:10.1001/jamanetworkopen.2026.0060)
Supplement: Supplement 2. — Data Sharing Statement [file jamanetwopen-e260060-s002.pdf]

Data Sharing Statement

Ceja. Preferred Panethnic Terms Among Latina/o and Hispanic Sexual and Gender Minority People. *JAMA Netw Open*. Published February 26, 2026.
doi:10.1001/jamanetworkopen.2026.0060

Data
Data available: No

Additional Information
Explanation for why data not available: The datasets generated during and/or analyzed during the current study are not publicly available due to ethical restrictions related to sensitive participant information but are available from the corresponding author on reasonable request. Researchers interested in The PRIDE Study data may submit a brief application which is reviewed by both a Research Advisory Committee (composed of scientists) and Participant Advisory Committee (composed of participants) to affirm appropriate data use. Details about the Ancillary Study process are available at www.pridestudy.org/collaborate or by contacting support@pridestudy.org or 855-421-9991 (toll-free).
